# The sustainable impact of an educational approach to improve the appropriateness of laboratory test orders in the ICU

**DOI:** 10.1371/journal.pone.0214802

**Published:** 2019-05-01

**Authors:** Benjamin Clouzeau, Marie Caujolle, Aurelie San-Miguel, Jerome Pillot, Nathalie Gazeau, Christophe Tacaille, Vincent Dousset, Fabienne Bazin, Frederic Vargas, Gilles Hilbert, Mathieu Molimard, Didier Gruson, Alexandre Boyer

**Affiliations:** 1 Department of Intensive Care Medicine, Pellegrin Hospital, Bordeaux, France; 2 Economic and Financial Department, Pellegrin Hospital, Bordeaux, France; 3 Departments of Laboratories, Pellegrin Hospital, Bordeaux, France; 4 Department of Radiology, Pellegrin Hospital, Bordeaux, France; 5 INSERM, U657 Pharmaco-Epidémiologie et Evaluation de l'Impact des Produits de Santé sur les Populations, France; University of Notre Dame Australia, AUSTRALIA

## Abstract

**Introduction:**

Few studies described strategies to improve the use of diagnostic tests in intensive care units (ICU). No study assessed whether their impact was sustained or not. In this study, we assessed whether a multi-faceted intervention for more appropriate use of laboratory testing can decrease the number of tests, is sustainable, is not associated with additional morbidity and represents a potential cost saving.

**Material and methods:**

An open-label prospective cohort study in two separated units of the same medical intensive care unit (ICU) including respectively 3315 and 2392 consecutive patients. After the observation period (2010), a reduction in ICU A of unnecessary diagnostics tests as part of a program including senior supervisory of juniors’ orders, encouragements for orders containment at each everyday round discussions (period 2; 2011). Period 3 (2012) consisted in the prolongation of the protocol as a routine care without supervision; Period 4 (2013) was a new period of observation without intervention. No modification was implemented in ICU B in periods 2–4.

**Results:**

After the intervention, a decrease in the overall number of tests per ICU-patient-days (37.3±5.5 (baseline) to 15.2±3.2 (- 59%); p<0.0001) was observed. The total cost of the tests decreased from 239±41 to 104±28 euros per ICU-patient days; p<0.0001. The effect on laboratory test orders was sustainable in period 3 (-49%) and 4 (-30%). No significant secondary effect of the intervention was observed in period 2. In ICU B, there was no significant change in the overall laboratory test orders in between the periods.

**Conclusions:**

Laboratory test containment is effective, likely safe and sustainable provided that an educational program is repeatedly promoted, that it makes sense for the whole team, that senior and junior physicians are both committed in the program, and that encouragements for laboratory orders containment at each everyday round discussions.

## Introduction

Improving patient health care is a priority. This can be done by improving quality and not necessarily quantity. A large proportion of hospital spending is constituted by laboratory and radiological tests [1]. These tests are generally overused in hospitals [2–3]. Because of both budget deficits and increase of health care expenditures [4–5], efforts are welcome to reduce unnecessary medical costs while providing high-quality health care. It has been estimated that 30% of computed tomography [6] or up to 20% laboratory tests may be unnecessary [7]. Decreasing the demands of unnecessary tests should be an everyday life task especially when they do not impact the care of patients.

The intensive care units (ICUs) do not escape this phenomenon. Most patients are monitored with several laboratory tests each day. Chemistry and haematology tests constitute 10% of total costs [1]. This could be explained by the severity of disease, the ease of blood drawing from indwelling catheters, the difficulty of implementing sustainable changes in a multidisciplinary environment and the absence of guidelines defining which frequency of routine laboratory tests is adequate [8–10]. Excessive use of laboratory blood tests increases resource utilization. It also contributes to blood loss and patient’s discomfort, and may eventually lead to improper diagnosis and treatment [1,11–13].

Few studies have described strategies to improve the use of diagnostic tests [14–17]. Most of them described the short term economic impact of a protocol. To our knowledge, no study assessed whether this short term impact was sustained or not.

Beyond the economic issue, the educational aspect of the appropriate use of laboratory tests is crucial. In line with this educational perspective, we developed a new multi-faceted intervention for more appropriate use of laboratory testing in our medical ICU with the goal of limiting orders to only those necessary laboratory tests. We hypothesized that the introduction of new guidelines for ordering laboratory tests in an ICU could significantly decrease the number of tests in a safe and sustainable way.

## Material and methods

### Setting

The study was carried out in two 12-bed ICUs (ICU A and ICU B) of the same medical department in a University teaching Hospital. Both ICUs have the same case mix but function as independent units without any mutual medical staff. The protocol was willingly only introduced and supervised in ICU A, ICU B serving as control. Three residents, one junior physician and three senior physicians worked in each ICU without difference in senior physicians’ age, gender and years of ICU practice. The residents left the ICU every 6 months. The junior physician moved from one unit to the other every one year. In ICU A, the educational program relied on two senior physicians (CB and BA) and two residents (CM and SMA, each a semester).

### Patients

All consecutive patients between January 1, 2010 and December 31, 2012, then from June 1, 2013 to December 31, 2013 were enrolled in the study. Demographic information included age, patient’s severity of illness score (SAPS II) and ventilation requirement [18].

### Periods of the study (Fig 1)

Period 1 (2010). For both ICUs, a first observation period was defined to determine the baseline number of laboratory tests which were ordered by the medical staff (mainly residents but also senior physicians). The total number of each laboratory tests was retrospectively calculated by administrators.

**Fig 1:**
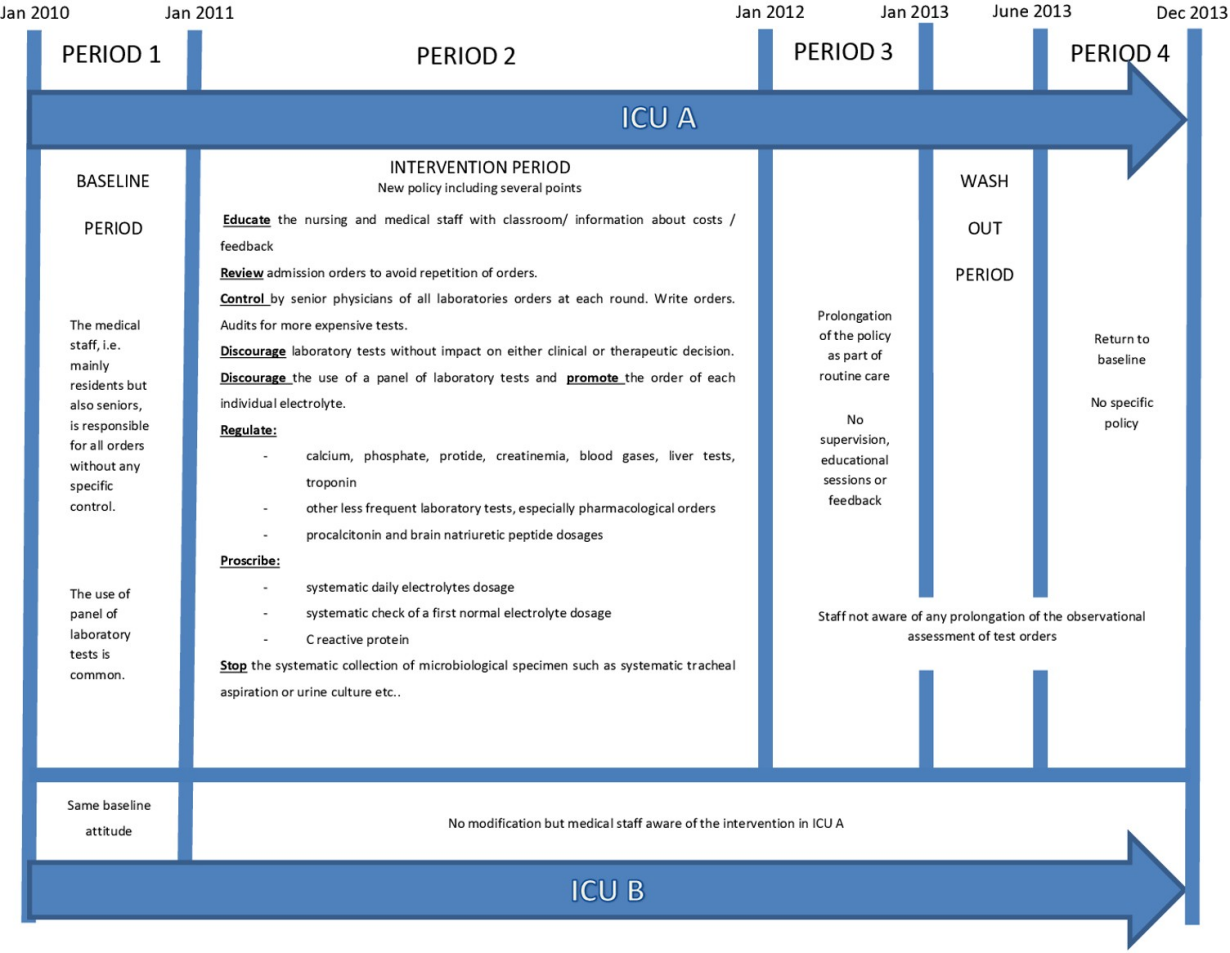
Characteristics of interventions according to different periods.

Period 2 (2011). The ICU A team decided to implement the intervention. Reduction of unnecessary diagnostics tests was part of an educational program including several points which were written in a prescribing guide (Fig 1). Our new policy included encouragements to reduce orders rather than penalty in case of over prescription since we were concerned that too much strictness may actually lead to more adverse events.

The medical staff of the ICU B was aware of this program and could feel free to implement it. This second unit was considered as the “control unit”, even if they could also apply the protocol. No medical staff was shared between units A and B during this year.

Period 3 (2012). This period consisted in prolonging the protocol as part of routine care without supervision, educational sessions or feedback. However, educational key messages on laboratory test orders merged into the entire junior physicians’ educational package. Laboratory orders were not challenged during the daily visits by senior physicians. The written guide was not available.

Period 4 (last 7 months of 2013). After a washout period of 4 months, this period was defined as the “reality period” i.e. a new period of observation without intervention as was done in the initial period 1. ICU B served as the control without any interventions or implementations in periods 2–4.

During period 3 and 4, the staff was not aware of any prolongation of the observational assessment of test orders. This was decided to remain as close as possible from the routine of the ICUs.

### Measurements

Financial administrators (GN, LC) of the Laboratory Department provided us with all monthly data concerning ICU orders (no individual data was provided). From the central database, they extracted the number of patients, number of laboratory tests and their respective costs.

For each unit (A or B), within each period (from period 1 to period 4), the reported results include the number of patients included, the number of patient-days (sum of patients X the mean length of ICU stay of the unit in which the patient has been admitted), the global number of tests ordered in each unit, the number of tests ordered per patient (sum of the tests / sum of patients), the number of tests per ICU-patient days (sum of the tests / sum of the patient-days).

Biochemical tests include all tests described in Table 1.

**Table 1:** Routine demand of main biochemical laboratory tests according to different periods.

Laboratory tests^1^	Period 1^2^ 2010	Period 2 2011	Period 3 2012	Period 4 2013
Creatinin	1,3	0,77	0,84	1,1
BUN	1,34	0,81	1,02	1,1
Potassium	1,35	1,08	1,43	1,42
Calcium	1,34	0,28	0,40	0,43
Glucose	1,34	0,22	0,24	0,40
Total Bilirubin	0,75	0,27	0,39	0,69
Sodium	1,35	0,97	1,16	1,29
Protide	1,35	0,23	0,29	0,43
Phosphore	1,34	0,28	0,19	0,43
CRP	0,10	0,03	0,02	0,02
BNP	0,20	0,02	0,02	0,02
PCT	0,65	0,10	0,06	0,07
Transaminases	0,56	0,22	0,3	0,46
GGT	0,43	0,18	0,3	0,46
Troponin	0,47	0,16	0,31	0,43
Blood count	1	0.74	0.89	1.12
Coagulation factors	0.41	0.28	0.29	0.38
Fibrinogen	0.33	0.35	0.40	0.53

^1^ Results are expressed as the overall number of test per ICU-patient days (where the ICU-patient days represent the sum of all days of all patients hospitalized in the ICU during the period).

^2^ All the tests comparing period 2,3 or 4 with period 1 were significant with p value <0.0001 except Total bilirubin and Troponin (period 4 vs. period 1;p = 0.6), CRP (period 4 vs. period 3;p = 0.06), and Firbinogen (period 3 vs. period 1;p = 0;7).

BUN blood urea nitrogen; CRP C reactive protein; BNP Brain natriuretic peptide; PCT Procalcitonin; GGT Gammaglutamyl transpeptidase

Haematological tests include coagulation tests and blood count. Immunological tests mainly include a battery of antibodies, complement, Coomb’s test, T cell subpopulations. Pharmacological tests are limited to serum drug dosage. The cost of biological tests and their evolution during the study periods are presented in Table 2.

**Table 2:** Cost^£^ of biological tests.

	Period 1 2010		Period 2 2011		Period 3 2012		Period 4 2013	
	euros	US dollars	euros	US dollars	euros	US dollars	euros	US dollars
Creatinin	2.16	2.44	1.89	2.14	1.89	2.14	1.89	2.14
BUN	2.16	2.44	1.89	2.14	1.89	2.14	1.89	2.14
Potassium	2.16	2.44	1.89	2.14	1.89	2.14	1.89	2.14
Calcium	2.16	2.44	1.89	2.14	1.89	2.14	1.89	2.14
Glucose	1.35	1.53	1.35	1.53	1.35	1.53	1.35	1.53
Total Bilirubin	3.24	3.66	2.7	3.05	2.7	3.05	2.7	3.05
Sodium	1.89	2.14	1.89	2.14	1.89	2.14	1.89	2.14
Protide	2.16	2.44	1.89	2.14	1.89	2.14	1.89	2.14
CRP	5.67	6.41	5.4	6.1	4.05	4.58	3.24	3.66
BNP	22.95	25.93	22.95	25.93	22.95	25.93	22.95	25.93
PCT	27	30.51	24.3	27.46	21.6	24.41	21.6	24.41
Transaminases	5.4	6.1	4.05	4.58	3.78	4.27	2.97	3.36
GGT	2.7	3.05	2.16	2.44	1.89	2.14	1.89	2.14
Gazometry	22.95	25.93	21.6	24.41	20.25	22.88	20.25	22.88
Troponin	17.55	19.83	17.55	19.83	17.55	19.83	17.55	19.83
Blood count	9.18	10.37	8.64	9.76	8.37	9.46	8.37	9.46
Coagulation factors	6.75	7.63	6.75	7.63	6.75	7.63	6.75	7.63
Fibrinogen	5.4	6.1	5.4	6.1	5.4	6.1	5.4	6.1

^**£**^ Current exchange rate: 1 Euro = 1.13 US Dollars

### End points

The primary end point is the reduction in the number of laboratory tests ordered in period 2 vs. 1

Secondary endpoints include the consecutive decrease in costs associated with test orders, the comparison of number of tests in ICU A within all periods. Providing that ICU B was the control unit for the baseline period, the number of tests in ICU A was compared with ICU B within all subsequent periods. Also, potential secondary effects of the reduction in the monitoring of laboratory tests were prospectively assessed during the intervention phase (twice a day at morning and afternoon round) and registered by the whole team (junior and senior physicians) in period 2 and only by senior physicians thereafter.

They were defined by clinically significant consequences of metabolic disorders (cardiac arrhythmia or change in electrocardiographic registration prompting an urgent correction of potassium or calcium, convulsive states related to calcium or sodium disorders).

### Ethics statement

The study was approved by ethics committee of the Société de Réanimation de Langue Française (n°CE SRLF 13–18).

### Statistical analysis

Ordinal and continuous variables with a normal distribution are expressed as mean ± SD. One-way analysis of variances (ANOVA) or Kruskall-Wallis when appropriate, were performed to study the significance between groups over different periods. The p values were two-sided, and the level of significance was set at < 0.05. Given the very high number of patients, which gives the study the power to identify very small and not clinically relevant differences between periods, we willingly decided not to report p value for clinical characteristics presented in Tables 3 and 4 (e.g. 1 point of SAPS II does not reflect any real clinical difference between two patients).

**Table 3:** Demographic characteristics according to different periods.

Laboratory tests	Baseline Period 1 2010	Protocol Period 2 2011	Routine Period 3 2012	Reality Period 4 2013^1^
N patients	875	883	983	574
ICU-patient days	4138	4433	4680	2327
Age	59.0 ± 3.2	59.5 ± 2.3	58.9 ± 3.2*	58.2 ± 0.7
Rate of mechanical ventilation	64.9 ± 7.4	63.5 ± 9.1	58.0 ± 10.8	64.5 ± 7.1**
SAPS II	52.6 ± 2.8	53.2 ± 3.2	53.7 ± 4.8	50.8 ± 1.3
Mortality rate ^£^	22	20	21.5	19

^1^ Last seven months; All the tests comparing pairs of period were significant with p <0.001 except:

*p = 0.41 period 3 vs 1

**p = 0.39 period 4 vs 1 and p = 0.04 period 4 vs 2

^£^ no statistical difference between periods (p = 0.47)

**Table 4:** Number and type of laboratory tests ordered in ICU A according to different periods.

Laboratory tests	Baseline Period 1 2010	Protocol Period 2 2011	Routine Period 3 2012	Reality Period 4 2013^1^
Biochemical tests				
N tests	135 120	53 463	74 692	47 271
N/N patients	154.4±29.8	60.5±14.2	76.0±7.8	82.4±12.7
N/ICU-patient days	32.7±5.9	12.1±3.4	16.0±10.7	20.3±3.1
Haematological tests				
N tests	15 185	10 608	12 843	10 348
N/N patients	17.4±4.2	12.0±2.1	13.1±1.8	18.0±3.1
N/ICU-patient days	3.7±0.8	2.4±0.4	2.7±2.0	4.4±0.9
Pharmacological tests				
N tests	1560	1265	1569	920
N/N patients	1.8±0.5	1.4±0.3	1.6±0.4	1.6±0.3*
N/ICU-patient days	0.4±0.1	0.3±0.1	0.3±0.3	0.4±0.1
Immunological tests				
N tests	517	655	530	552
N/N patients	0.6±0.5	0.7±0.4	0.5±0.3	1.0±0.5
N/ICU-patient days	0.1±0.1	0.1±0.1	0.1±0.1	0.2±0.1
Virological tests				
N tests	1786	1194	1477	1304
N/N patients	2.0±0.8	1.4±0.5	1.5±0.4	2.3±0.9
N/ICU-patient days	0.4±0.2	0.3±0.1	0.3±0.2	0.6±0.2
Mycoparasitological tests				
N tests	66	67	95	421
N/N patients	0.1±0.1***	0.1±0.0**	0.1±0.1****	0.7±0.5
N/ICU-patient days	0.0±0.0	0.0±0.0**	0.0±0.0	0.2±0.1
Overall number of tests per ICU-patient days	37.3±5.5	15.2±3.2	19.3±13.2	26.0±4.1
Total Euros per ICU-patient days ^2^	238.9 ±40.6	103.9 ±27.9	145.2±99.6	181.9 ±28.8

^1^ Last seven months

^2^ The cost does not include immunological and mycoparasitological tests

All the comparisons yielded significant differences with p<0.001 except

*no statistically significant difference between P4 and P3

** no statistically significant difference between P1 and P2

*** no statistically significant difference between P1 and P3

**** no statistically significant difference between P2 and P3

N number of test orders; ICU Intensive care unit; SAPS Simplified acute physiology score

## Results

During the study, a total of 3315 patients were admitted to ICU A and 2392 in ICU B.

### Period 1

The orders of all laboratory tests are summarized in Table 1. Biochemical tests accounted for the vast majority of the overall orders (86–88%). At baseline, despite not similar, the number of laboratory tests was close between the two medical ICUs, with a difference of no more than 10–20 tests/patient.

### Endpoints

No clinically relevant difference in age, SAPS II and number of mechanically ventilated patients was observed in ICU A within different periods (Table 3).

#### Primary endpoint (Tables 4 and 5 and Fig 2)

After the intervention (period 2), the overall number of laboratory test per ICU-patient days decreased in ICU A from 37.3±5.5 (baseline) to 15.2±3.2 (- 59.2%) p<0.0001 (Table 4), leading to a cost reduction (239±41 baseline vs 104±28 period 2 (euros per ICU-patient days) p<0.0001) (Table 5).

**Fig 2:**
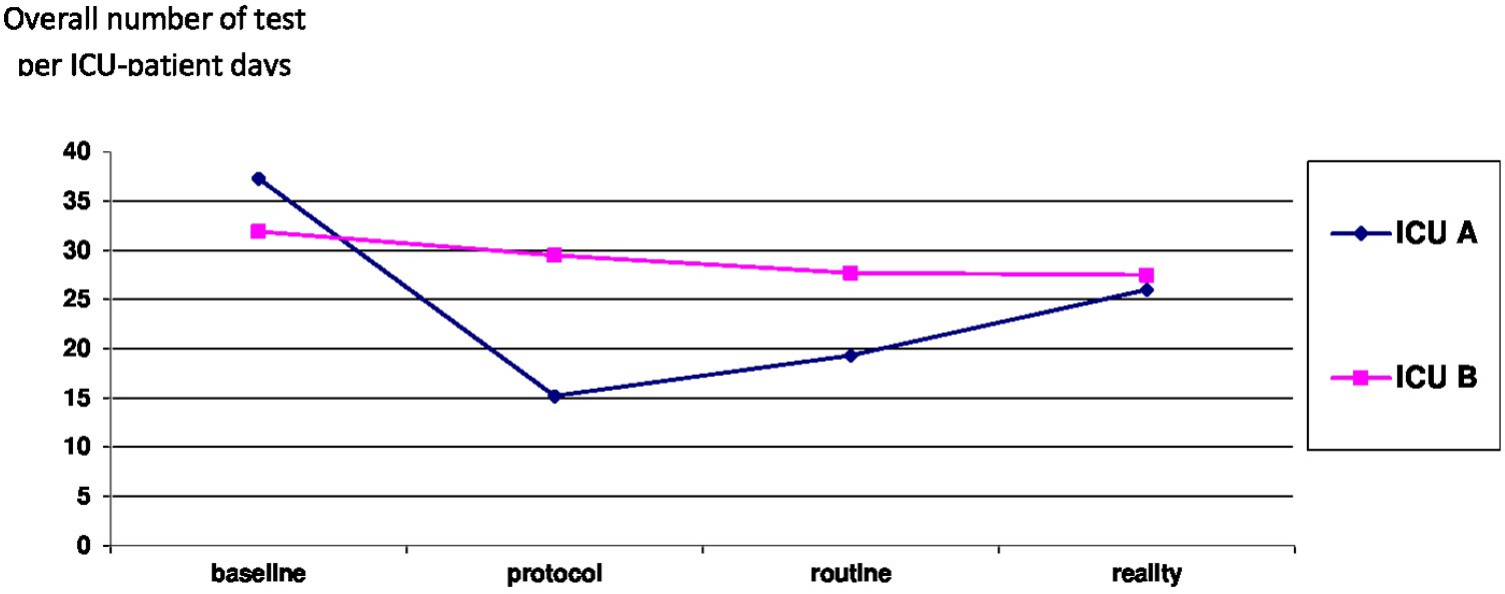
Comparison of the number of test per ICU-patient days between ICU A and B.

**Table 5:** Comparison of laboratory tests costs in ICU A according to different periods.

	Euros^£^ per ICU-patient days				
Laboratory tests	Period 1, 2010	Period 2, 2011	Period 3, 2012	Period 4, 2013	p*
Biochemical	182.6 ±35.5	64.9 ±26.9	96.2 ±61.5	113.5 ±16.0	<0.0001
Haematological	27.0 ± 6.2	18.1 ± 2.8	23.8 ±19.3	32.5 ± 6.2	<0.0001
Pharmacological	8.3 ± 2.5	5.6 ± 1.1	7.1 ± 6.7	8.3 ± 1.6	<0.0001
Virological	20.9 ± 7.2	15.3 ± 7.9	18.1 ±13.1	27.6 ±10.4	<0.0001
Total	238.9 ±40.6	103.9 ±27.9	145.2 ±99.6	181.9 ±28.8	<0.0001

^**£**^ Current exchange rate: 1 Euro = 1.13 US Dollars

* Kruskall Wallis test: the p value refers to the comparison test of the costs during any period vs. any other periods

#### Other endpoints

Comparison within periods into ICU A. A decrease in the overall number of tests per patients [176 at baseline vs 76 in period 2 (- 57%)] was observed. The reduction was more pronounced regarding biochemical tests (-60%), which accounted for 88% of the total laboratory orders at baseline vs. 80% in period 2. The total cost of the tests decreased from 1.005.805 euros to 503.551 euros (-50%). The ratio of the overall number of tests per patient-ICU days increased by 27% in period 3 vs. period 2 but remained lower than baseline (- 48%). This relapse was accompanied by a total increase in cost of 39% between period 2 and 3.

Two years after baseline, during period 4, the daily demands of laboratories tests increased in comparison with period 3 (+35%), even if they still remained lower than baseline (-30%).

No significant adverse event of the intervention was observed in 2011. No difference in rates of mortality, transfer of patients from our ICU to cardiac intensive care unit (a marker for delay in myocardial infarction diagnosis) was observed in period 2 vs. 1.

Comparison between ICU A and B (Table 6 and Fig 2). In ICU B, there was no real impact of the protocol implemented in ICU A despite the proximity of both units. Considering the entire study period, the overall laboratory test orders decreased slowly from 31.9 to 27.5 tests per ICU-patient days.

**Table 6:** Comparison of laboratory test orders between ICU A and B according to different periods^1^.

Laboratory tests	Period 1		Period 2		Period 3		Period 4	
	ICU A	ICU B	ICU A	ICU B	ICU A	ICU B	ICU A	ICU B
N patients	875	675	883	634	983	658	574	425
N ICU patients days	4138	3972	4433	4139	4680	4274	2327	2440
Biochemical tests								
N tests	135 120	109464	53 463	104 737	74 692	101 620	47 271	53838
N/N patients	154.4	162.2	60.5	165.2	76.0	154.4	82.4	126.7
N/N ICU-patient days	32.7	27.6	12.1	25.3	16.0	23.7	20.3	22.1
Haematological tests								
N tests	15 185	13 789	10 608	14 194	12 843	13 725	10 348	10291
N/N patients	17.4	20.4	12.0	22.4	13.1	20.9	18.0	24.2
N/N ICU-patient days	3.7	3.5	2.4	3.4	2.7	3.2	4.4	4.2
Pharmacological tests								
N tests	1560	1629	1265	1505	1569	1677	920	883
N/N patients	1.8	2.4	1.4	2.4	1.6	2.5	1.6	2.1
N/N ICU-patient days	0.4	0.4	0.3	0.4	0.3	0.4	0.4	0.4
Immunological tests								
N tests	517	282	655	613	530	460	552*	539*
N/N patients	0.6	0.4	0.7	1.0	0.5	0.7	1.0	1.3
N/N ICU-patient days	0.1	0.1	0.1	0.1	0.1	0.1	0.2	0.2
Virological tests								
N tests	1786	1463	1194	1588	1477	1282	1304	1129
N/N patients	2.0	2.2	1.4	2.5	1.5	1.9	2.3	2.7
N/N ICU-patient days	0.4	0.4	0.3	0.4	0.3	0.3	0.6	0.5
N Mycological tests								
N tests	66	57	67	90	95	72	421	278
N/N patients	0.1	0.1	0.1	0.1	0.1	0.1	0.7	0.7
N/N ICU-patient days	0.0	0.0	0.0	0.0	0.0	0.0	0.2	0.1
Overall tests per patient	176.3	187.7	76.1	183.6	92.8	180.5	106	157.7
Overall tests per ICU-patient days	37.3	31.9	15.2	29.5	19.3	27.7	26.0	27.5
Total Euros per ICU-patient days	239	218	104	206	145	184	182	193

^1^ All statistical tests have been used to compare ICU A vs ICU B within each period: they were all statistically significant with p<0.0001, except

*p = 0.0526

ICU Intensive care unit

## Discussion

Identifying unnecessary tests can be a complex and challenging task since some tests remain essential for screening, diagnosis, and monitoring of critical diseases. It is complex first because ICU patients are critically ill and second because of the different level of experience among physicians. This could explain a large number of tests not directly contributing to the process of care decision. We have shown that the introduction of few simple procedures in a medical ICU led to a significant and sustained reduction in unnecessary diagnostic and monitoring tests. Thereby, we achieved a major cost reduction. Whether this reduction could result from demographic or case-mix difference between periods is not sustained by our data despite statistically significant differences. Given the very high number of patients included in the study, even minuscule difference between periods could indeed result in statistical difference (e.g. 1 point of SAPS II does not reflect any real clinical difference between the patients of two different periods). In contrast, the global cost reduction could be partly explained by the evolution of costs year after year.

We decided to unbundle the classic panel tests, such as the “ionogram”. A special attention for each classic electrolyte (inexpensive when taken aside) should be relevant for the overall cost reduction. Larsson et al. calculated that 5% of total costs in clinical chemistry in Sweden could be saved based on seven of the most frequently used tests [19]. In our study, there was a decrease in the number of orders in all the components of the laboratory, even if the results were more pronounced in the biochemistry subunit.

Several interventions to improve physicians testing practices have been previously described [19–21]. One recent study showed a 28% reduction in test orders in the intervention vs. pre-intervention period which persisted one year after the intervention [22] but no previous report estimated the mid-term sustainability of this approach. The sustainability of the intervention effect was partly lost in period 4 vs period 2,3. Even if the difference remained relevant (- 30% in overall number of tests per ICU-patient days), it is highly plausible that a reasonable objective of test ordering in our ICU should be set between 33.2 and 14.6 tests per ICU patient days. One could believe that the observed lost in sustainability over time could increase. However, the main driving force of sustainability is the change in cultural paradigm represented by the following issues.

- **Who?** It is likely much more challenging to implement changes, especially to observe sustainability over time, with more providers coming-and-going as well as greater variations in practice patterns. Therefore, particularly if many providers are involved, it is important to identify in the staff a single physician committed in test orders at the bedside, ensuing the prototype of pharmacist-assisted antibiotic prescriptions. This may be junior physicians’ role since they have a nonstop bedside activity.
- **With the support of whom?** Junior physician should be supported by older physicians with experience and academic teaching leadership. Even if senior physicians have been associated with a lower guideline compliance, their expertise may supplant the age factor to contribute to the real change we observed [23].
- **When?** Laboratory test orders should be discussed during the day shift at a specific time, i.e. at the morning and afternoon round, which precludes night providers to interfere with these orders.
- **Why?** To decrease laboratory test orders must make sense. If physicians (especially in younger ones [24]) generally pay more attention to expensive tests than to frequent and cheap tests, a previous study reported no impact on the amount of laboratory orders when physicians were informed of their cost [25]. We therefore did not focus our new approach on the financial aspect, but rather on moral values that make sense to physicians.
- **How?** Quarterly reported feedback to nursing and medical staff was performed but was not the key to sustainable change, instead of the content of educational sessions which is crucial. We insisted on the association between excessive use of laboratory blood tests and inappropriate resource utilization, patient’s discomfort, blood loss and improper diagnosis and treatment, as well as useless costs. Interestingly, a trade-off between an ambitious aim (period 2), i.e. achievable but not sustainable and a status quo is probably a factor of sustainability (-20% decrease as obtained in period 4 would be both achievable and sustainable).

We suspect that unnecessary tests were generally ordered by juniors residents in period 1. It is likely that residents want to apply freshly learned theoretical knowledge without distinguishing useful from futile tests [24]. Before period 2, the junior physician whose one role is to check for test orders could have been influenced by recently graduated residents. The educational aspect of the above-cited systematic questions questions “Do I really need it? etc.” is not sufficiently taught in books and academic lectures. In period 2, no such influence could occur since residents have to answer to a panel of questions.

Physicians of ICU B of the same department modified their behaviour regarding laboratory test ordering while just aware of the protocol implemented in the ICU next door. By their move from one unit to another every one year, junior physicians might have driven this spread.

Our study has some limitations. It only reflects the experience of a single center, and generalizability may be limited as a result of differences in case mix and hospital organization, particularly in units in which many providers are involved. Second, no data were collected about the potential savings in blood products. Third, we did not measure the feelings and confidence of physicians. Lastly, despite no increase in mortality rate occurred and despite complications due to a missed test were not observed, we cannot exclude that orders reduction could result in an unidentified delayed adverse effects such as delay in the diagnosis of myocardial infarction or acute kidney injury. However, concerning troponin dosage, no study has shown the clinical benefit of a systematic daily order. Had a clinically significant myocardial ischemia occurred, we could have diagnosed it via cardiac arrhythmias or fatal events, all outcomes carefully recorded during the study. Moreover, should a clinically relevant delay in myocardial infarction diagnosis and management have occurred, a higher proportion of patients undergoing percutaneous coronary intervention or surgical procedure could have been observed. This was not the case. Concerning acute kidney injury, blood urea and creatinin were ordered once a day except in special indications (such as a decision to initiate renal replacement therapy in which a second dosage the same day could be useful), which makes delay in recognizing renal dysfunction less likely.

## Conclusion

A policy of laboratory test orders containment could be effective, likely safe and sustainable, provided that the following points are organized. An educational program for test containment must make sense to everybody involved in the patient’s care. Senior and junior physicians must be both committed in the program. Encouragements and discussions for laboratory test orders containment must be included in the everyday round discussions. Despite no specifically reported data, we believe there was no increase in harm or diagnostic delays associated with this policy.

## Supporting information

S1 Dataset Boyer data. (XLS)
